# Evaluation of Wild, Wine, Table, and Raisin Grapevine (*Vitis* spp.) Genotypes in Gedeo Zone, Southern Ethiopia

**DOI:** 10.1155/2022/6852704

**Published:** 2022-01-29

**Authors:** Dargie Tsegay Berhe, Derbew Belew

**Affiliations:** ^1^Dilla University, College of Agriculture and Natural Resources, Dilla, Ethiopia; ^2^Jimma University, College of Agriculture and Veterinary Medicine, Jimma, Ethiopia

## Abstract

Grapevine is one of the major horticultural crops of the world with the cultivated area exceeding 7.5 million ha used for a myriad of products ranging through fresh table grape, preserves, juice, wine, and raisins. The main objective of this study was to introduce twenty-eight grapevine cultivars (ten wild, ten wine, four table, and four raisin grapes) into Gedeo Zone for the first time and ampelographically characterize them in Dilla and Yirgacheffe agroecological conditions in Gedeo Zone, Southern Ethiopia, from August 2018 to July 2021. Ten *Vitis abyssinica* wild grapevine cultivars were collected from Adama, Addis Ababa, Alamata, Arba Minch, Bahir Dar, Dire Dawa, Gondar, Hawassa, Jimma, and Jinka areas. Additional ten world class wine grapes were gathered from Ziway Castel Winery, and four table and four raisin grapes were also collected from Raya Horti Farm and Koka Vineyard at the same time. The experiment was a 2 × 28 factorial arranged in randomized complete block design (RCBD) with three replications, and data were analyzed using the R-software. The analysis of variance revealed that the interaction of cultivar and location significantly (*P* < 0.001) affected grapevine plant height, leaf number, number of fruits per plant, and tendril number per vine, while grapevine trunk diameter, flower cluster, root length, and number of suckers per vines were not significantly (*P* > 0.05) influenced by the interaction of the two factors. Generally, the wine grapevine cultivars had lower canopy such as plant height, leaf number, number of tendrils, and suckering vines while these registered a higher number of fruits per plant, trunk diameter, flower cluster, and root length compared to the wild grapevine cultivars. The results of the present study suggested that Syrah, Chenin Blanc, and Grenache can produce high grapevine berry yield and wine quality in Gedeo Zone agroecology particularly in Dilla location. The wild grapevines collected from Dire Dawa, Arba Minch, Jinka, and Alamata were the potential candidates for the world class wine, raisin, and table grapevines which could open new frontiers in the future for Ethiopian native *Vitis abyssinica* wild grapevine breeding and genetic engineering that will help to move the national and international viticulture and enology industry forward. As the Ethiopian native grapevines are at the risk of total extinction, adequate conservation strategies are required. Breeding, detailed identification, and introducing the potential grapes in different regions of the country are needed. This finding represents a step forward in efforts to understand hybridization of *Vitis abyssinica* grapevine with *Vitis* vinifera and other new world *Vitis* species.

## 1. Introduction

Viticulture is one of the major horticultural industries of the world [1] with the area of cultivation exceeding 7.5 million ha [2] and used for a myriad of products ranging through fresh table grape, preserves, juice, wine, and raisins [3, 4]. The grape attains a high concentration of sugar as well as a wide range of aromatic compounds when ripe. On the other hand, the presence of relatively high levels of acids means that the fruit is amenable to many different uses. Approximately 50% of global grape production is used for wine, 36% for table grapes, 8% for raisins, 5% for juice, and 1% to produce other products [5].

Globally, a complex of factors determines the success of a viticulture industry with climate being the dominant one [6]. For the successful production of grapes, the mean annual temperature is the most critical factor. Grapes require hot, dry summers and cool winters [7, 8]. An ideal site for vineyard must provide full sunlight [9], with access to good quality water throughout the growing season, protection from excessive winds, and no late spring frosts [10]. Grapes can be cultivated in a wide variety of soils including sandy loam, sandy clay loam, shallow to medium black soils and red loam but respond best to sandy loam soil. In addition, grapevines can grow successfully in a wide range of soil pH (4.0–9.5), but a range of 6.5–8.0 is ideal [11].

Grapevine has been grown in a few parts of Ethiopia since ancient times. Different biotic and abiotic stresses such as climate change, diseases, war, and change of frontiers have resulted in losing hundreds of Ethiopian native grapevine (*Vitis abyssinica*) cultivars. Yet, the Ethiopian native grapevines are not well registered and researched; instead, they remained as a wild plant and invasive weed [12]. The *Vitis abyssinica* native grapevines need to be restored, collected, and well registered that could open new frontiers for the future breeding and genetics in viticulture and enology industry. To the best of our knowledge, there is neither a single grapevine plant available nor any grapevine research conducted in Gedeo Zone. Therefore, the main goal of this study was to introduce the native wild grapevines and world class wine, raisin, and table grape cultivars to Gedeo Zone and characterize them in Dilla and Yirgacheffe agroecological conditions.

## 2. Materials and Methods

### 2.1. Description of the Experimental Sites

**Figure 1:**
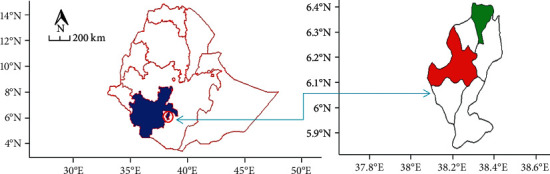
Research site (blue color = SNNPRS; red color = Yirgacheffe; green color = Dilla).

The experiment was carried out in Dilla, located at 6° 24′ 45″ N latitude and 38° 18′ 03″ E longitude, and in Yirgacheffe, located at 6° 09′ 43″ N latitude and 38° 12′ 21″ E longitude districts of Gedeo Zone, Southern Ethiopia (Figure 1) from August 2018 to July 2021. Dilla and Yirgacheffe have an altitude of 1434 and 1881 meters above sea level and are located at 361 and 399 km south of Addis Ababa, respectively.

### 2.2. Experimental Design and Treatments

The treatments were comprised of two research locations (Dilla and Yirgacheffe) and twenty-eight grapevines (Syrah, Malbec, Chardonnay, Merlot, Chenin Blanc, Sauvignon Blanc, Cabernet Sauvignon, Semillon, Pinot Noir, Grenache, Concord, Cardinal, Perlette, Sugraone, Ruby seedless, Thompson seedless, Flame seedless, Crimson seedless, Adama wild, Addis Ababa wild, Alamata wild, Arba Minch wild, Bahir Dar wild, Dire Dawa, Gondar wild, Hawassa wild, Jimma wild, and Jinka wild). The treatments were combined in a randomized complete block design (RCBD) factorial experiment, resulting in a total of 56 treatment combinations with three replications and of 168 total observations (2 ∗ 28 ∗ 3).

### 2.3. Experimental Procedures

Ten native wild grapevines were collected from Adama, Addis Ababa, Alamata, Arba Minch, Bahir Dar, Dire Dawa, Gondar, Hawassa, Jimma, and Jinka. At the same time, ten world class wine grapes (Syrah, Malbec, Chardonnay, Merlot, Chenin Blanc, Sauvignon Blanc, Cabernet Sauvignon, Semillon, Pinot Noir, and Grenache) were gathered from Ziway Castel Winery. Additional four table grapes (Concord, Cardinal, Perlette, Sugraone) and four raisin grapes (Ruby seedless, Thompson seedless, Flame seedless, Crimson seedless) were collected from Raya Horti Farm and Koka Vineyard. Healthy and a pencil-sized vine cuttings were used from one-year-old cane at dormancy period. After careful transporting, grapevine cuttings were cut with five buds each and planted in soil media with 50% top soil, 30% compost, and 20% sand for four months in 30 cm distance each. In their fifth month, the seedling was carefully pruned, roots managed, and transplanted into the well-prepared research sites. The spacings were 1.5 meters between plants and 2 meters between block. Wood poles were erected and wires stretched across the poles to support the vines and for proper grapevine cane and canopy management. All grapevine agronomic practices (watering, weeding, trellising, etc.) were kept in appropriate practice during grape production resulting in safe and healthy food while taking into account economic, social, and environmental sustainability.

### 2.4. Data Collection Procedures

Grapevine cultivars have been mainly characterized and identified by standard ampelographic descriptors. Samples of grapevines were randomly taken from each treatment for vegetative and yield related parameters. Data were collected for grapevine plant height, leaf number, fruit number per plant, root length, trunk diameter, number of suckers that emerged from the trunk, flower clusters per vine, and tendril numbers. Grapevine plant height was measured from the soil surface to the top most growth points of above ground plant part. Trunk diameter was measured at Veriason grapevine growth stage (north to south and east to west dimension of above ground grapevine trunk). The number of leaves, fruits, tendrils, and suckers per vine was counted and calculated as average. Root length as a pioneer variable for water and nutrient uptake was calculated by measuring the length of orthogonal and diagonal grapevine underground roots.

### 2.5. Statistical Analysis

The experiment was subjected to two-way analysis of variance in randomized complete block design and data were analyzed using the *R*-software (version 4.1.1, 2021). Analysis of variance was performed to determine the effect of the independent variables on the dependent parameters at the 5% significance level (*P* < 0.05). To determine the significant differences between treatment means, Fisher's range test was applied. Correlation analysis was also computed to record the relationship among the principal components.

## 3. Results and Discussion

### 3.1. Plant Height (m)

The interaction effect of cultivar and location on mean grapevine plant height was highly significant (*P* < 0.001). The highest plant heights were registered in the wild grapevines collected from Addis Ababa (2.87 ± 0.13) followed by wild grapevines collected from Jimma (2.78 ± 0.11) and Gondar (2.70 ± 0.07) grown in Dilla condition, while the least plant heights were recorded in Syrah in Yirgacheffe (0.60 ± 0.09) and Dilla (0.65 ± 0.06), followed by Chenin Blanc in Yirgacheffe (0.66 ± 0.12) and Dilla (0.76 ± 0.04) agroecology. Plant height in Addis Ababa wild grapevine was statistically higher by 79.09% than in Syrah wine grapevine (Table 1) in Yirgacheffe.

**Figure 2:**
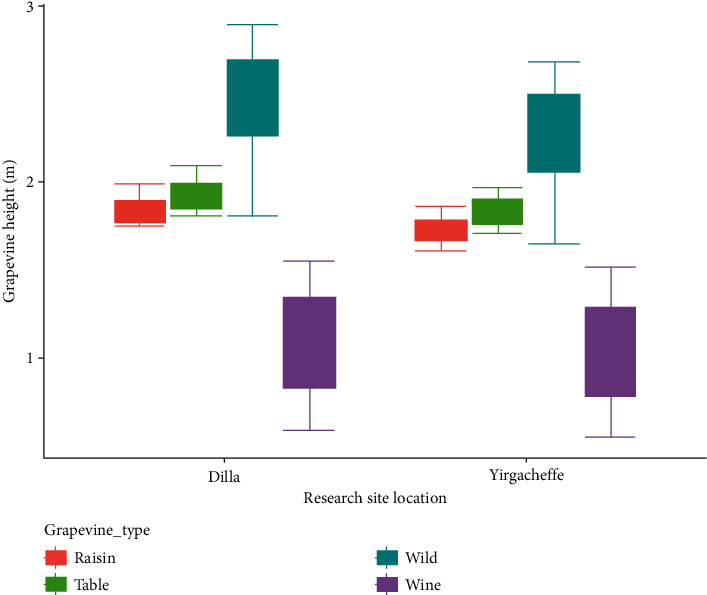
Response of plant height to grapevine cultivar types and research site locations.

The findings of this study succinctly illustrated that wild grapevines collected from Adama, Addis Ababa, Alamata, Arba Minch, Bahir Dar, Dire Dawa, Gondar, Hawassa, Jimma, and Jinka had significantly longer plant height in comparison with the world class table, raisin, and wine grapevines. However, the shorter plant heights were recorded in the most popular European (Pinot Noir, Grenache, Cabernet Sauvignon, Sauvignon Blanc, Semillon), Argentinian (Malbec), Australian (Syrah, Chenin Blanc), and American (Merlot, Chardonnay) wine grapes in both Dilla and Yirgacheffe agroecological conditions. The raisin and table grapevines (South African and European) had a moderate plant height compared to the wild and wine grapevines (Figure 2). The result of the present study is in line with [13, 14], that clearly and concisely reported that wild grapevines were vigorous compared to the modern grapes. This might be mainly due to genetic differences among the grapevine cultivars [15], sunlight distribution [9], and poor canopy management [16].

In the current study, it was observed that some wild grapevines could be best candidates of raisin and table grapes based on their plant height. For instance, the wild grapevine collected from Dire Dawa (1.68 ± 0.08) had statistically the same plant height as Crimson seedless raisin grapevine (1.68 ± 0.12) in Yirgacheffe condition. In line with this finding, Dire Dawa wild grapevine (1.84 ± 0.11) had a uniform plant height with Perlette table grape (1.84 ± 0.08) in Dilla condition and with Ruby seedless raisin grapevine (1.84 ± 0.02) in Yirgacheffe. Arba Minch wild grapevine (1.92 ± 0.14) could also be a potential candidate of table or raisin grapevine type which had similar plant height with Perlette raisin (1.95 ± 0.12) and cardinal table (1.87 ± 0.31) grapes in Yirgacheffe. On top of that, plant height of Jinka wild grapevine (2.06 ± 0.02) grown in Yirgacheffe was similar to Sugraone table grape (2.06 ± 0.08) in Dilla condition. In this regard, [17] identified that wild grapevines were used to produce new grapevines resistant to rootstock diseases, drought tolerant, high yield, and best quality through hybridization. Generally, shorter grapevines are recommended in the viticulture and wine industry. This might be due to pruning and training at the right time and in a proper system as a core element to produce high berry yield and make thus wine of good quality [18].

### 3.2. Trunk Diameter (cm)

**Figure 3:**
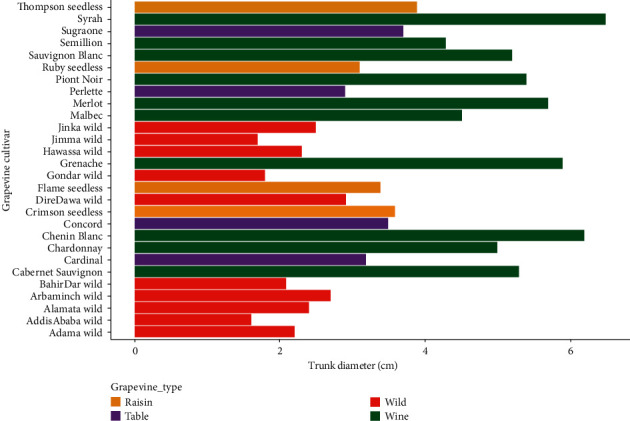
Trunk diameter in grapevine cultivars and grapevine types.

The main effect of grapevine cultivars on trunk diameter showed a highly significant (*P* < 0.001) variation, while the interaction effect of cultivar and location was not significant (*P* > 0.05) (Table 2). The highest trunk diameter was registered in Syrah (6.23 ± 0.27) followed by Chenin Blanc (5.83 ± 0.29) and Grenache (5.65 ± 0.23), while the lowest trunk diameter was registered in the wild grapevine cultivars collected from Addis Ababa (1.40 ± 0.19) followed by Jimma (1.50 ± 0.19) and Gondar (1.55 ± 0.21) areas. Syrah wine grapevine was thicker by 77.53% than the wild grapevine collected from Addis Ababa. From the wild grapevines, only Dire Dawa wild (2.60 ± 0.30) was a potential candidate for table grape that had statistically the same trunk diameter with Perlette (2.62 ± 0.17) (Figure 3).

**Figure 4:**
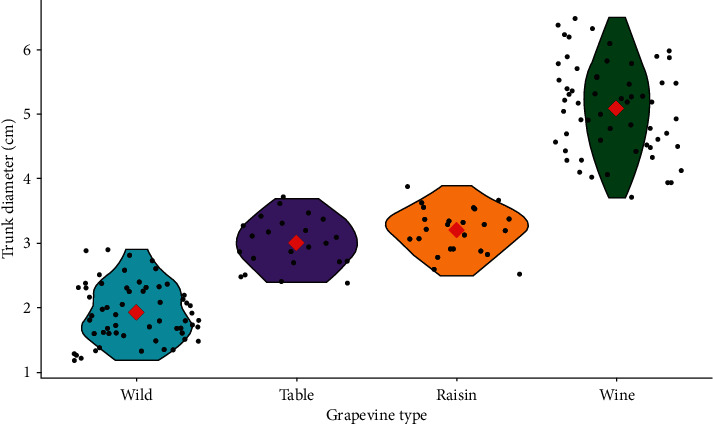
Trunk diameter variation among grapevine types.

Most of the raisin grapes had statistically the same trunk diameter as the table grapes. For instance, trunk diameter of Concord table grape (3.15 ± 0.25) was identical with Flame seedless raisin cultivar (3.13 ± 0.22), Sugraone table grape (3.40 ± 0.23) with Crimson seedless raisin grape (3.34 ± 0.19), and Cardinal table grape (2.88 ± 0.26) with Ruby seedless raisin grapevine (2.78 ± 0.24). Basically, wine grapes had significantly higher trunk diameter while the minimum values were recorded in the wild grapevines (Figure 4).

The results of this study are in line with [19] that adequately explored that grapevine trunk diameter varied according to variety. This might be due to the genetic differences and trunk capacity to absorb water and minerals [15].

**Figure 5:**
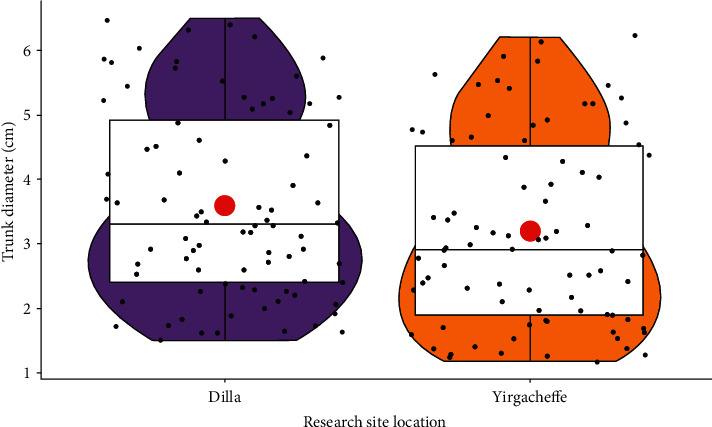
Grapevine trunk diameter in different growth locations.

The influence of location in mean trunk diameter showed highly significant (*P* < 0.001) difference. All grapevines grown in Dilla condition had notably higher trunk diameter than grapevines grown in Yirgacheffe agroecology. Apparently, trunk diameter in Dilla (3.60 ± 1.44) was higher by 11.11% than grapevines grown in Yirgacheffe (3.20 ± 1.45) condition (Figure 5). This could be due to the environmental factors as confirmed by [20, 21] that attested that vine trunk diameter was directly influenced by the agroecological site variations.

### 3.3. Root Length (cm)

The main effect of grapevine cultivars on root length showed highly significant differences (*P* < 0.001), whereas the interaction effect of cultivar and location was not significant (*P* > 0.05). The longest roots were observed in Syrah (134.73 ± 4.23), Chenin Blanc (125.43 ± 4.62), and Grenache (119.18 ± 3.11), while the shortest roots were found in the wild grapevine cultivars collected from Addis Ababa (27.38 ± 2.18), Jimma (28.53 ± 1.94), and Gondar (31.28 ± 2.26) areas. Syrah scored longer root by 79.68% than the wild grapevine collected from Addis Ababa. Dire Dawa wild (55.98 ± 2.53) was a candidate for table grape as it had the same root length with the Perlette (2.62 ± 0.17) cultivar (Table 2).

**Figure 6:**
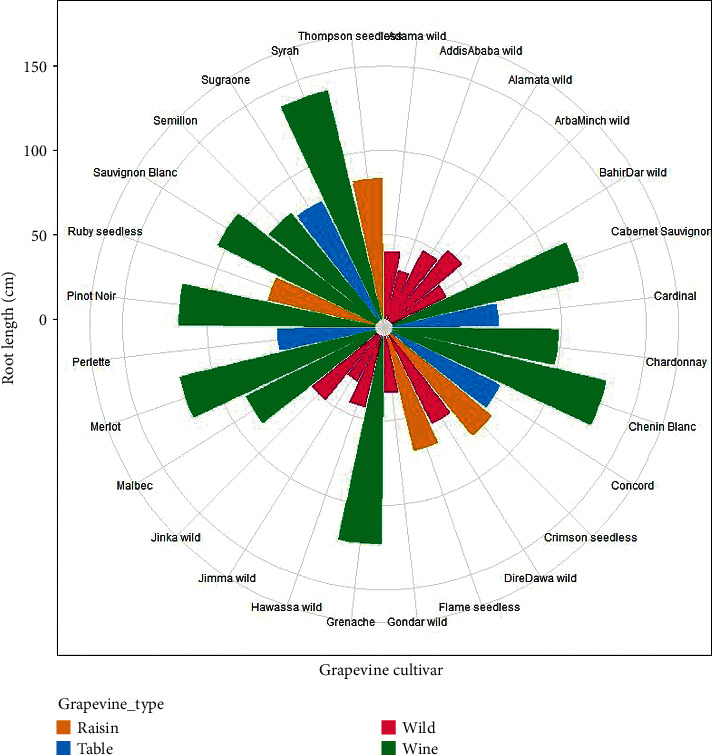
Grapevine root lengths among grapevine cultivars and grape types.

Most of the raisin grapes were alike in root length with the table grapevines. For instance, root length in Concord table grape (67.45 ± 2.93) was the same as Flame seedless raisin cultivar (67.11 ± 2.75); and Sugraone table grape (74.30 ± 3.49) with Crimson seedless (73.53 ± 3.61) raisin grape. In terms of grapevine types, wine grapes had significantly higher root length, while the minimum values were recorded in the wild grapevines. The raisin and table grapevine cultivars had lower root length compared to the wine grapes but higher root length in comparison with the wild grapevines (Figure 6). In line with the current findings, [22, 23] identified that grapevine cultivars have quite various root lengths and diversified shapes. This might be due to genetic and/or environmental factors influencing on the grapevine root dynamics and pattern [23], root genotype differences, root development and dry matter partitioning, root system, root morphology, root formation, and distribution [24, 25].

The influence of location difference in the mean root length of grapevines showed highly significant variations (*P* < 0.001). All grapevines grown in Dilla condition had notably longer root than grapevines grown in Yirgacheffe agroecology. Accordingly, the root length in Dilla (74.24 ± 31.81) was higher by 6.99% than grapevines grown in Yirgacheffe (69.05 ± 31.14) condition (Figure 6) that could be due to genetic or environmental factors. In this regard, [22, 23] reported that environmental and genetic factors affect the grapevine root size and depth in different locations.

### 3.4. Number of Fruits per Plant

The interaction effect of cultivar and grapevine growth location on mean number of fruits per plant showed significant differences (*P* < 0.05). The maximum number of fruits per vine was observed in Syrah (295.76 ± 7.57) and Chenin Blanc (283.59 ± 11.24) grown in Dilla while the minimum number of fruits was recorded in the wild grapevines collected from Addis Ababa grown in Yirgacheffe (16.02 ± 2.06) and Dilla (19.99 ± 2.00) followed by Jimma wild grapevine in Yirgacheffe (24.51 ± 4.73) and Dilla (27.62 ± 4.16) along with Gondar wild grapevine grown in Yirgacheffe (31.79 ± 2.52) and Dilla (36.02 ± 3.04) conditions. The typical Australian Syrah red wine grape in Dilla agroecology scored a higher number of fruits by 94.58% than Addis Ababa wild grapevine grown in Yirgacheffe (Table 1).

**Figure 7:**
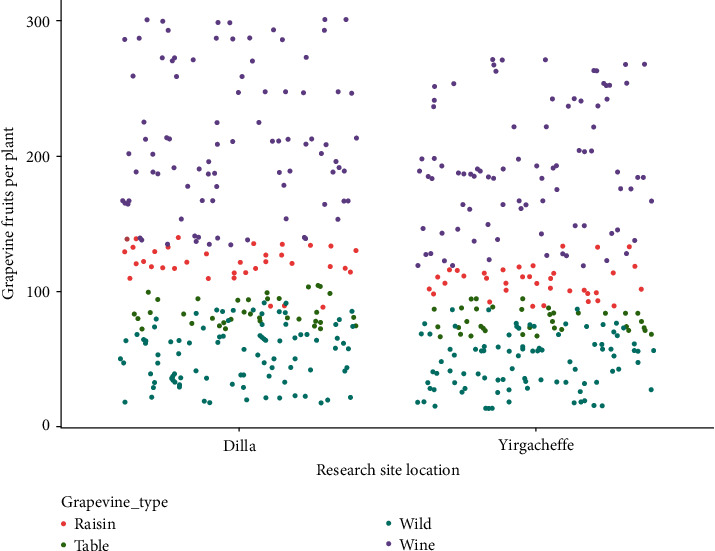
Response of fruit number to grapevine types and growth locations.

At a glance, the highest number of fruits per plant was recorded in wine grapevines compared to those in the raisin, table, and wild grapevines grown in both Dilla and Yirgacheffe. The raisin and table grapevines had higher fruit number compared to the wild grapevine cultivars but lower than the wine grapevine cultivars (Figure 7). This is in accordance with [21, 26] that reported that wild grapevine cultivars had lower number of fruits per plant, smaller berry size, and weak berry development patterns compared to the modern wine, table, and raisin grape cultivars. This might be due to genetic variations [15], poor canopy management, and vigorous nature of wild grapevine species [13, 14].

The data presented above vividly depicts that some wild grapevine cultivars had statistically equal fruit number with potential raisin and table grape cultivars. Just to mention, wild grapevine cultivars collected from Dire Dawa grown in Yirgacheffe (79.07 ± 7.03) had statistically the same number of fruits per vine as Concord in Dilla (84.67 ± 8.96) and Yirgacheffe (78.18 ± 8.72), Cardinal in Dilla (85.38 ± 8.50) and Yirgacheffe (78.83 ± 5.03), Perlette in Dilla (76.23 ± 4.16), and Yirgacheffe (68.67 ± 2.09) as well as Sugraone in Dilla (88.79 ± 5.69) agroecology. The number of fruits per plant in the wild grapevine cultivars collected from Arba Minch and Jinka was also the same as Concord, Cardinal, and Perlette table grapevine cultivars grown in both Dilla and Yirgacheffe conditions. In the same way, Alamata wild grapevine cultivar had statistically equal number of fruits per vine with Concord and Cardinal table grapes in Yirgacheffe and with Perlette grapevine in Dilla condition. This result is in consistence with the study of [21] that evaluated wild grapevine and found potential candidates for the modern grapevine breeding that could move the vine and wine industry forward. Generally, the higher fruits per vine was found in the shorter wine grapes while the lower fruit number was recorded in the wild grapevine with long vine height which was supported by [17].

### 3.5. Number of Leaves per Vine

The interaction effect of cultivar and location on grapevine leaf number showed highly significant (*P* < 0.001) differences. The maximum leaf number was observed in the wild grapevines collected from Addis Ababa grown in Dilla (164.38 ± 1.53) and Yirgacheffe (160.12 ± 3.60) followed by wild grapevines collected from Jimma (158.38 ± 1.50) and Gondar (152.37 ± 1.53) both grown in Dilla while the minimum leaf number was recorded in Syrah grapevine (30.77 ± 1.60) and Chenin Blanc (33.68 ± 2.08) grown in Yirgacheffe followed by Syrah grapevine grown in Dilla (38.08 ± 2.01) and Grenache in Yirgacheffe (39.40 ± 1.49). The leaf number per vine in Addis Ababa wild grapevine grown in Dilla condition was statistically higher by 81.28% than Syrah wine grapevine in Yirgacheffe (Table 1).

**Figure 8:**
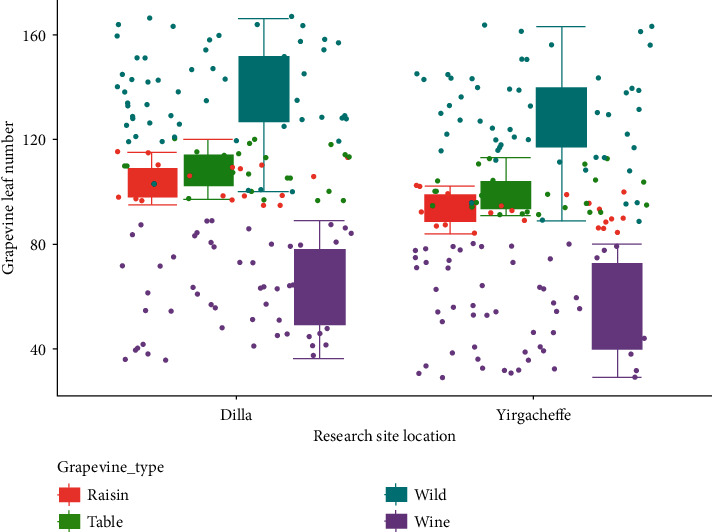
Response of leaf number to grapevine types and locations.

The findings of this study concisely determined that wild grapevines had significantly higher leaf number per vine while lower leaf numbers were recorded in wine grapevines grown in Dilla and Yirgacheffe agroecological conditions. The raisin and table grapes had a moderate leaf number per vine compared to the wild and wine grapevines (Figure 8). This is in line with [15] that succinctly reported that wild grapevines had huge leaf number and vigorous canopy compared to modern grapevines. This could be mainly due to genetic variations among cultivars and poor canopy management [15]. Sometimes, leaf removal [27] and shoot, cluster, or bunch thinning [28] were suggested as a compulsory agronomic practice to keep the vine healthy and well productive and improve chemical and sensory wine quality [29]. According to [27], vine physiology, berry development, and wine quality were significantly influenced by the timing of grapevine leaf removal.

In this study, it was observed that some wild grapevines had similar leaf number to some raisin and table grapes. The wild grapevine collected from Dire Dawa (93.42 ± 3.79) had statistically the same leaf number as Sugraone table grapevine (91.84 ± 0.58) grown in Yirgacheffe. In line with this, Dire Dawa wild grapevine grown in Dilla (101.41 ± 1.55) had an equal leaf number with Cardinal table grape (101.03 ± 2.65) and Ruby seedless raisin grapevine (100.53 ± 1.50) in Yirgacheffe condition and with Flame seedless raisin grapevine (101.11 ± 4.36) in Dilla. Likely, Arba Minch wild grapevine grown in Yirgacheffe (111.04 ± 2.65) had similar leaf number with Perlette raisin in Yirgacheffe (109.43 ± 4.73), cardinal table (112.39 ± 2.08), and Thomson seedless raisin (112.69 ± 2.48) grapevines in Dilla agroecology. Indeed, leaf number of Jinka wild grapevine (117.21 ± 1.10) grown in Yirgacheffe was also similar to Perlette table grape (117.69 ± 2.52) grown in Dilla condition. In this regard, [13] investigated that wild grapevine had similar vine physiology with modern grapevine cultivars. According to [17], excess leaf number per plant causes several vine diseases, weak berry development, small berry size, and poor wine quality.

### 3.6. Flower Cluster per Plant

The grapevine flower cluster per plant showed a highly significant variation (*P* < 0.001) among cultivars while the interaction of effect of cultivar and location was not significant (*P* > 0.05). The highest number of flower clusters was observed in Syrah (16.32 ± 2.07) and Chenin Blanc (15.31 ± 1.75), while the lowest was registered in the wild grapevine cultivars collected from Addis Ababa (1.17 ± 0.41) and Jimma (1.49 ± 0.55) areas. The number of flower cluster per plant in Syrah wine grapevine was higher by 92.83% than the wild grapevine collected from Addis Ababa (Table 2).

**Figure 9:**
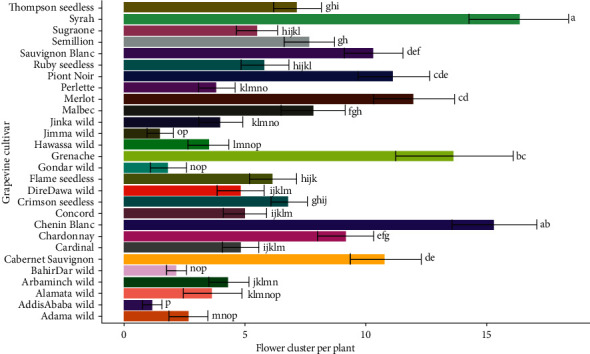
Response of flower cluster number to grapevine cultivars.

The wild grapevines collected from Dire Dawa, Arba Minch, and Jinka were potential candidates for a table and raisin grapes. As a confirmation, Dire Dawa (4.86 ± 0.98), Araba Minch (4.34 ± 0.82), and Jinka (4.04 ± 0.89) wild grapevines had statistically the same number of flower cluster per vine as the table grapevines of Cardinal (4.85 ± 0.75), Concord (5.02 ± 0.89), and Perlette (3.84 ± 0.75). From the raisin grapevines, Ruby seedless (5.81 ± 0.98) had an equal number of flower cluster with the Dire Dawa wild grapevine (Figure 9).

**Figure 10:**
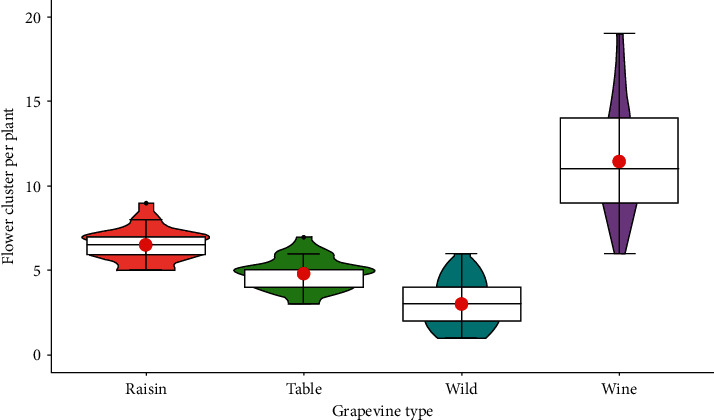
Response of flower cluster number to grapevine types.

In terms of grapevine types, wine grapes had significantly higher number of flower cluster per plant while the minimum values were observed in the wild grapevines. The raisin and table grapevine cultivars had a lower number of flower clusters than the wine grapes but a higher number compared to the wild grape. Accordingly, some wild grapevines, specially those collected from Dire Dawa, Jinka, Arba Minch, and Alamata areas, were similar in the number of flower cluster to some world-class wine, raisin, and table grapevines (Figure 10).

**Figure 11:**
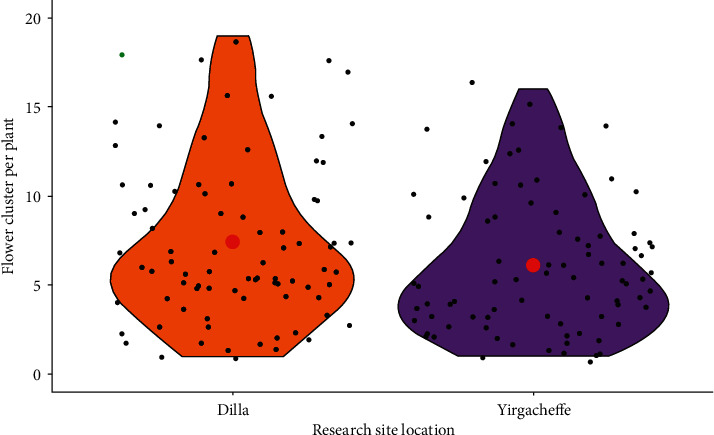
Grapevine flower cluster number in different research site locations.

The influence of location variation in the mean number of flower cluster per vine showed also highly significant differences (*P* < 0.001). All grapevines grown in Dilla condition had notably higher number of flower cluster than grapevines grown in Yirgacheffe. Apparently, the number of flower clusters per vine in Dilla (7.40 ± 4.57) was higher by 17.43% than grapevines grown in Yirgacheffe (6.11 ± 3.85) agroecological condition (Figure 11). This finding was in line with [9, 30] that reported that wild grapevines had enormous leaf number compared to modern grapevines. This might be mainly due to genetic variations among cultivars [15], shoot thinning, timing and intensity of elevated temperatures [8], impact of defoliation, temperature conditions at budburst, the extent of primary branching, girdling of shoots, and pollen viability [31].

This is in accordance with the findings of [19] that reported that flower number varied from location to location that might be mainly due to temperature fluctuations [7, 8], vine phenology [6], and seasonal variations in grapevine yield components based on pre- and post-flowering weather conditions [32].

### 3.7. Number of Tendrils per Vine

The interaction effect of cultivar and grapevine growth location on mean tendril number was highly significant (*P* < 0.05). The maximum number of tendrils per vine was observed in the wild grapevines collected from Addis Ababa and grown in Dilla condition (25.69 ± 0.58) followed by wild grapevines collected from Jimma (24.43 ± 0.54) grown in Dilla while the minimum number of tendrils was recorded in Syrah grapevine (4.87 ± 0.59) and Chenin Blanc (5.14 ± 1.09) both grown in Yirgacheffe. Addis Ababa wild grapevine had statistically higher tendril number by 81.04% than Syrah wine grapevine grown in Yirgacheffe (Table 1).

**Figure 12:**
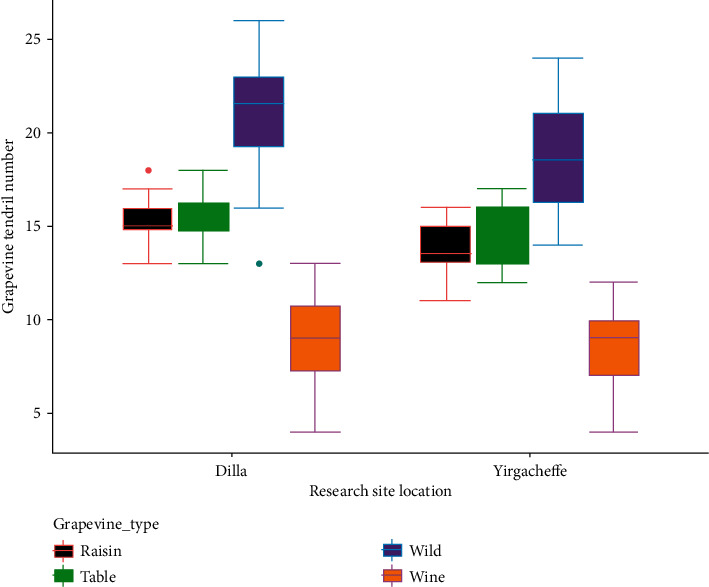
Response of tendril number to grapevine cultivar types and research site locations.

The findings of this study indicated that wild grapevines had significantly higher tendril number while the lowest number of tendrils was recorded in wine grapevines grown in both Dilla and Yirgacheffe. The raisin and table grapevines had higher tendril number compared to the wine grapevines but lower than the wild grapevine cultivars (Figure 12). Similar research findings were reported by [13–15] who found significant tendril number differences among grapevine cultivars. This might be due to genetic variations [15], vigorous nature of the vine [13], temperature [7], sunlight distribution [9], and canopy management [16].

In the current study, it was observed that some wild grapevine cultivars had statistically equal number of tendrils per vine with some potential raisin and table grape cultivars. For instance, wild grapevine cultivars collected from Dire Dawa grown in Yirgacheffe (15.17 ± 1.12) and Dilla (15.03 ± 1.73) had statistically the same number of tendrils per vine with Crimson seedless grown in Dilla (14.68 ± 0.60), and Yirgacheffe (13.64 ± 1.46), Ruby seedless (14.74 ± 1.53), Flame seedless (14.08 ± 0.89), Sugraone (14.10 ± 1.11) in Yirgacheffe, and Thompson seedless (14.12 ± 1.11) in Dilla. On top of this, Jinka (16.39 ± 1.52) and Arba Minch (16.18 ± 1.09) wild grapevine grown in Yirgacheffe had statistically the same tendril number with Ruby seedless (17.11 ± 1.21) in Dilla as well as Sugraone (16.68 ± 1.55), Concord (16.00 ± 1.99), and Cardinal (15.81 ± 1.57) table grape grown in Yirgacheffe condition. The number of tendrils per vine in Alamata wild grapevine grown in Yirgacheffe (17.56 ± 1.60) was the same as Ruby seedless, Sugraone, Concord in Dilla, and Cardinal in Yirgacheffe. On the same trend, several researchers' findings [13, 17, 18] indicated that there was great possibility of selecting a native wild grapevine to produce new grapevines that could be resistant and tolerant to grapevine biotic and abiotic stresses, respectively.

### 3.8. Number of Suckers

The number of suckers that emerged from each grapevine trunk was significantly influenced (*P* < 0.001) by cultivar, but the interaction effect of cultivar and location was not significant (*P* > 0.05). The highest number of suckers was registered in the wild grapevine cultivars collected from Addis Ababa (3.17 ± 1.17), Jimma (2.83 ± 1.17), Bahir Dar (2.69 ± 0.81), and Gondar (2.69 ± 1.21). On the other hand, minimum number of suckers was observed in Sugraone (0.18 ± 0.41), Pinot Noir (0.52 ± 0.54), Cabernet Sauvignon (0.50 ± 0.55), and Crimson seedless (0.67 ± 0.52) grapevines. Fortunately, there were not any suckers recorded in the world class wine grapes of Syrah, Merlot, Grenache, and Chenin Blanc and in the potential raisin grapevine of Thompson seedless (Table 2).

**Figure 13:**
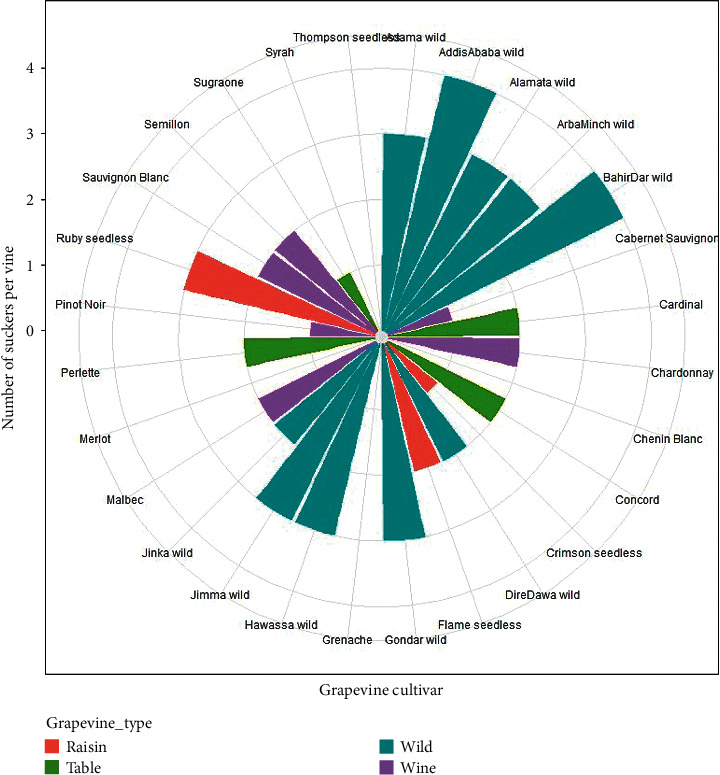
Number of grapevine suckers in different cultivars and grapevine types.

From the wild grapevines, only Dire Dawa wild (1.50 ± 0.55) had statistically the same number of suckers as Perlette (1.50 ± 0.57), Cardinal (1.18 ± 0.41), and Concord (1.00 ± 0.63) table grapes; as Semillon (1.52 ± 0.52), Malbec (1.33 ± 0.52), and Sauvignon Blanc (0.83 ± 0.75) wine grapes; and as Ruby seedless (1.36 ± 1.21) and Flame seedless (1.17 ± 0.98) raisin grapes. Indeed, most of the table grapes had similar number of suckers to some wine and raisin grapes. For instance, the number of suckers per vine in Perlette table grape was statistically equal to the number of suckers in Semillon, Malbec, Chardonnay, and Sauvignon Blanc wine grapes, as well as to Ruby seedless and Flame seedless raisin grapes (Figure 13).

In terms of grapevine types, wild grapevine cultivars had a significantly higher number of suckers while the minimum values were recorded in the wine grapevines. The raisin and table grapevine cultivars had a higher number of suckers compared to the wine grapevines but lower suckers in comparison with the wild grapevine cultivars collected from different areas of the country. Similar research findings were reported by [13, 14, 17, 33], that found a higher number of suckers in wild grapevines than in wine, raisin, and table grapevine cultivars. This finding is possibly attributed to genetic factors [15] and/or poor canopy management [16].

The influence of location difference in the mean number of grapevine suckers showed a highly significant variation (*P* < 0.001). Mainly the raisin grapevines grown in Dilla condition had notably lower number of suckers than those grown in Yirgacheffe agroecology (Table 3). Accordingly, the number of suckers in Dilla (1.08 ± 0.93) was lower by 25.52% than grapevines grown in Yirgacheffe (1.45 ± 1.26). This is in accordance with the findings of [33] that reported that the number of suckers per vine varied from location to location that might be due to temperature fluctuations [7] and water deficit [34].

## 4. Conclusion and Recommendations

Grapevine is one of the major horticultural crops used for a myriad of products such as preserves, vinegar, oil, juice, table grape, raisins, and wine. Even though the Ethiopian agroecology is suitable for producing various grapevine cultivars, there was not any grapevine plant or research trial in Gedeo Zone using worldwide or wild grapevines. The Ethiopian native grapevines (*Vitis abyssinica*) were ignored since the Italian invasion and considered as a weed plant. For this research, ten native grapevines were collected from different areas in Amhara, Tigray, Oromia, SNNPRS, and Sidama regions and used to characterize them in comparison with some world-class wine, grape, and table grapes. In this context, the findings of the study indicated that Syrah, Chenin Blanc, and Grenache wine grapes were the potential cultivars for high berry yield and wine of good quality in Gedeo Zone agroecology, mainly in Dilla location. Accordingly, Dire Dawa, Arba Minch, Jinka, and Alamata wild grapevines were the best candidates for raisin and table grapevines.

As the Ethiopian native grapevines are at the risk of total extinction, adequate conservation strategies are required. Breeding, detailed identification of Ethiopian wild grapevines, and introducing the potential wine grapes to different regions of the country are also expected. Therefore, the present work represents a step forward in the efforts to understand the hybridization of *Vitis abyssinica* grapevine with *Vitis vinifera* and/or other new world *Vitis* species.

## Figures and Tables

**Table 1:** Interaction effect of cultivar and location on grapevine plant height, the number of leaves, tendrils, and fruit per plant.

Cultivar	Location	Plant height	Leaf number	Tendril number	Fruit number
Adama wild	Dilla	2.46 ± 0.07^fg^	140.05 ± 2.04^e^	22.10 ± 0.98^cde^	51.04 ± 3.00^wxy^
Addis Ababa wild	Dilla	2.87 ± 0.13^a^	164.38 ± 1.53^a^	25.69 ± 0.58^a^	19.99 ± 2.00^BC^
Alamata wild	Dilla	2.38 ± 0.05^h^	128.74 ± 0.58^gh^	20.77 ± 0.59^ef^	68.02 ± 5.29^stuv^
Arba Minch wild	Dilla	2.17 ± 0.09^j^	120.06 ± 1.20^ij^	17.71 ± 1.53^ghijk^	75.29 ± 11.02^rst^
Bahir Dar wild	Dilla	2.54 ± 0.06^de^	145.04 ± 1.99^d^	22.65 ± 0.61^bcd^	41.32 ± 3.06^yz^
Cabernet Sauvignon	Dilla	1.17 ± 0.08^yz^	65.38 ± 1.48^x^	9.07 ± 1.10^wxy^	199.41 ± 12.34^de^
Cardinal	Dilla	1.85 ± 0.10^mn^	112.39 ± 2.08^k^	15.34 ± 1.15^mnop^	85.38 ± 8.50^pqr^
Chardonnay	Dilla	1.34 ± 0.12^uv^	78.07 ± 2.65^v^	11.07 ± 1.24^tuv^	170.12 ± 7.01^g^
Chenin Blanc	Dilla	0.76 ± 0.04^E^	42.69 ± 2.11^B^	6.00 ± 0.84^ABCD^	283.59 ± 11.24^a^
Concord	Dilla	1.96 ± 0.08^l^	105.05 ± 1.89^m^	16.00 ± 1.99^klmno^	84.67 ± 8.96^pqr^
Crimson seedless	Dilla	1.79 + 0.04^opq^	106.08 ± 2.97^lm^	14.68 ± 0.60^opqr^	128.71 ± 7.09^ijk^
Dire Dawa wild	Dilla	1.84 ± 0.11^nop^	101.41 ± 1.55^n^	15.03 ± 1.73^nopq^	87.33 ± 4.16^pqr^
Flame seedless	Dilla	1.85 ± 0.03^no^	101.11 ± 4.36^n^	15.35 ± 0.64^mnop^	118.08 ± 4.03^klm^
Gondar wild	Dilla	2.70 ± 0.07^c^	152.37 ± 1.53^c^	23.11 ± 0.87^bc^	36.02 ± 3.04^zA^
Grenache	Dilla	0.83 ± 0.05^D^	48.35 ± 2.55^A^	6.72 ± 1.59^zABC^	260.00 ± 12.53^b^
Hawassa wild	Dilla	2.42 ± 0.11^gh^	134.04 ± 1.01^f^	21.20 ± 0.88^def^	62.35 ± 4.51^uvw^
Jimma wild	Dilla	2.78 ± 0.11^b^	158.38 ± 1.50^b^	24.43 ± 0.54^ab^	27.62 ± 4.16^ABC^
Jinka wild	Dilla	2.26 ± 0.05^i^	126.36 ± 1.49^h^	18.51 + 2.08^ghi^	70.24 ± 8.19^stu^
Malbec	Dilla	1.40 ± 0.13^u^	82.76 ± 1.54^u^	11.13 + 0.97^tuv^	153.81 ± 13.01^h^
Merlot	Dilla	1.01 ± 0.07^B^	55.33 ± 1.48^yz^	8.46 + 0.59^xyz^	227.01 ± 19.08^c^
Perlette	Dilla	1.84 ± 0.08^nop^	117.69 ± 2.52^ij^	14.79 + 1.51^opqr^	76.23 ± 4.16^qrs^
Pinot Noir	Dilla	1.11 ± 0.14^zA^	62.36 ± 1.15^x^	9.14 + 1.03^wxy^	208.69 ± 5.86^d^
Ruby seedless	Dilla	1.98 ± 0.22^l^	96.67 ± 1.59^op^	17.11 + 1.21^hijklm^	105.77 ± 14.98^mno^
Sauvignon Blanc	Dilla	1.23 ± 0.05^wx^	72.03 ± 1.09^w^	10.09 + 1.14^uvwx^	188.91 ± 2.08^ef^
Semillon	Dilla	1.52 ± 0.03^t^	87.34 ± 1.53^st^	12.10 + 0.96^st^	137.68 ± 2.52^i^
Sugraone	Dilla	2.06 ± 0.08^k^	98.05 ± 1.73^no^	16.68 + 1.55^ijklmn^	95.63 ± 10.97^op^
Syrah	Dilla	0.65 ± 0.06^F^	38.08 ± 2.01^C^	5.39 + 1.15^BCD^	295.76 ± 7.57^a^
Thompson seedless	Dilla	1.76 ± 0.02^q^	112.69 ± 2.48^k^	14.12 + 1.11^pqr^	133.10 ± 6.12^ij^

Adama wild	Yirgacheffee	2.29 ± 0.05^i^	131.71 ± 1.61^fg^	19.44 ± 0.68^fg^	44.09 ± 3.61^xyz^
Addis Ababa wild	Yirgacheffee	2.66 ± 0.13^c^	160.12 ± 3.60^b^	23.25 ± 1.10^bc^	16.02 ± 2.06^C^
Alamata wild	Yirgacheffee	2.18 ± 0.07^j^	121.08 ± 1.04^i^	17.56 ± 1.60^hijkl^	60.69 ± 7.23^uvw^
Arba Minch wild	Yirgacheffee	1.92 ± 0.14^lm^	111.04 ± 2.65^k^	16.18 ± 1.09^klmno^	67.85 ± 8.50s^tuv^
Bahir Dar wild	Yirgacheffee	2.23 ± 0.19^i^	138.37 ± 2.08^e^	18.82 ± 2.12^gh^	36.24 ± 3.09^zA^
Cabernet sauvignon	Yirgacheffee	1.12 ± 0.23^yz^	57.33 ± 1.60^y^	9.60 ± 0.78^vwxy^	182.44 ± 5.67^fg^
Cardinal	Yirgacheffee	1.87 ± 0.31^mn^	101.03 ± 2.65^n^	15.81 ± 1.57^lmnop^	78.83 ± 5.03^qrs^
Chardonnay	Yirgacheffee	1.29 ± 0.23^vw^	72.73 ± 1.57^w^	10.49 ± 0.68^tuvw^	151.19 ± 9.17^h^
Chenin Blanc	Yirgacheffee	0.66 ± 0.12^F^	33.68 ± 2.08^D^	5.14 ± 1.09^CD^	253.15 ± 10.53^b^
Concord	Yirgacheffee	1.78 ± 0.15^pq^	95.04 ± 1.06^opq^	13.20 ± 0.94^rs^	78.18 ± 8.72^qrs^
Crimson seedless	Yirgacheffee	1.68 ± 0.12^rs^	90.36 ± 1.58^rs^	13.64 ± 1.46^qrs^	113.43 ± 3.06^lmn^
Dire Dawa wild	Yirgacheffee	1.68 ± 0.08^rs^	93.42 ± 3.79^pqr^	15.17 ± 1.12^nopq^	79.07 ± 7.03^qrs^
Flame seedless	Yirgacheffee	1.76 ± 0.16^q^	95.80 ± 3.06^op^	14.08 ± 0.89^pqr^	102.10 ± 3.61^no^
Gondar wild	Yirgacheffee	2.50 ± 0.08^ef^	140.21 ± 2.65^e^	20.91 ± 0.63^ef^	31.79 ± 2.52^zAB^
Grenache	Yirgacheffee	0.77 ± 0.12^DE^	39.40 ± 1.49^BC^	7.10 ± 1.08^zAB^	233.53 ± 10.02^c^
Hawassa wild	Yirgacheffee	2.23 ± 0.13^ij^	126.83 ± 2.52^h^	18.16 ± 1.05^ghij^	56.69 ± 4.04^vwx^
Jimma wild	Yirgacheffee	2.58 ± 0.08^d^	146.52 ± 4.16^d^	21.79 ± 0.58^cde^	24.51 ± 4.73^ABC^
Jinka wild	Yirgacheffee	2.06 ± 0.02^k^	117.21 ± 1.10^j^	16.39 ± 1.52^jklmno^	63.81 ± 9.54^tuvw^
Malbec	Yirgacheffee	1.32 ± 0.07^v^	77.38 ± 2.08^v^	11.21 ± 1.10^tuv^	134.51 ± 13.87^i^
Merlot	Yirgacheffee	0.92 ± 0.06^C^	46.78 ± 3.06^A^	7.76 ± 0.67^yzA^	197.99 ± 6.51^de^
Perlette	Yirgacheffee	1.95 ± 0.12^l^	109.43 ± 4.73^kl^	15.45 ± 1.24^mnop^	68.67 ± 2.09^stuv^
Pinot Noir	Yirgacheffee	1.05 ± 0.06^AB^	53.81 ± 0.58^z^	8.53 ± 0.54^xyz^	189.20 ± 4.02^ef^
Ruby seedless	Yirgacheffee	1.84 ± 0.02^nop^	100.53 ± 1.50^n^	14.74 ± 1.53^opqr^	94.75 ± 6.66^op^
Sauvignon Blanc	Yirgacheffee	1.18 ± 0.21^xy^	64.44 ± 1.61^x^	9.69 ± 0.68^vwx^	172.28 ± 11.93^g^
Semillon	Yirgacheffee	1.46 ± 0.05^t^	79.23 ± 1.08^v^	11.87 ± 0.58^stu^	126.11 ± 2.65^ijkl^
Sugraone	Yirgacheffee	1.73 ± 0.12^qr^	91.84 ± 0.58^qr^	14.10 ± 1.11^pqr^	88.79 ± 5.69^pq^
Syrah	Yirgacheffee	0.60 ± 0.09^F^	30.77 ± 1.60^D^	4.87 ± 0.59^D^	263.90 ± 10.21^b^
Thompson seedless	Yirgacheffee	1.63 ± 0.13^s^	85.69 ± 1.53^tu^	13.22 ± 2.03^rs^	121.20 ± 11.14^jkl^

Mean		1.74	97.51	14.39	118.15
CV		2.33	2.22	8.00	6.73
LSD		0.07	3.49	1.86	12.86
*P* value		^*∗∗∗*^	^*∗∗∗*^	^*∗∗*^	^*∗*^

Means within a column followed by the same letter(s) are not significantly different at 5% LSD test (^*∗∗∗*^*P* < 0.001, ^*∗∗*^*P* < 0.01, ^*∗*^*P* < 0.05, and ns = *P* > 0.05).

**Table 2:** The main effect of cultivars on grapevine trunk diameter, root length, flower number, and number of suckers per plant.

Cultivar	Flower cluster	Trunk diameter	Root length	Suckers
Adama wild	2.67 ± 0.82^no^	1.87 ± 0.27^s^	37.33 ± 2.90^s^	2.33 ± 0.52^bcd^
Addis Ababa wild	1.17 ± 0.41^p^	1.40 ± 0.19^u^	27.38 ± 2.18^v^	3.17 ± 1.17^a^
Alamata wild	3.66 ± 1.21^mn^	2.07 ± 0.28^qr^	43.80 ± 2.67^q^	2.00 ± 0.63^cde^
Arba Minch wild	4.34 ± 0.82^klm^	2.42 ± 0.25^p^	51.98 ± 2.57^o^	1.67 ± 0.82^defg^
Bahir Dar wild	2.18 ± 0.43^op^	1.78 ± 0.24^s^	34.20 ± 1.97^t^	2.69 ± 0.81a^bc^
Cabernet Sauvignon	10.83 ± 1.47^d^	5.00 ± 0.27^f^	109.27 ± 5.39^e^	0.50 ± 0.55^jkl^
Cardinal	4.85 ± 0.75^jkl^	2.88 ± 0.26^n^	58.38 ± 3.22^m^	1.18 ± 0.41^fghij^
Chardonnay	9.19 ± 1.17^e^	4.62 ± 0.25^h^	92.27 ± 3.73^g^	1.16 ± 0.43^fghij^
Chenin Blanc	15.31 ± 1.75^a^	5.83 ± 0.29^b^	125.43 ± 4.62^b^	0.00 ± 0.00^l^
Concord	5.02 ± 0.89^jk^	3.15 ± 0.25^m^	67.45 ± 2.93^k^	1.00 ± 0.63^ghij^
Crimson seedless	6.85 ± 0.75^fgh^	3.34 ± 0.19^l^	73.53 ± 3.61^j^	0.67 ± 0.52^ijkl^
Dire Dawa wild	4.86 ± 0.98^jkl^	2.60 ± 0.30^o^	55.98 ± 2.53^n^	1.50 ± 0.55^efgh^
Flame seedless	6.17 ± 0.98^ghi^	3.13 ± 0.22^m^	67.11 ± 2.75^k^	1.17 ± 0.98^fghij^
Gondar wild	1.87 ± 0.75^op^	1.55 ± 0.21^t^	31.28 ± 2.26^u^	2.69 ± 1.21^abc^
Grenache	13.69 ± 2.42^b^	5.65 ± 0.23^c^	119.18 ± 3.11^c^	0.00 ± 0.00^l^
Hawassa wild	3.50 ± 0.84^mn^	2.00 ± 0.33^r^	40.52 ± 2.68^r^	2.00 ± 0.89^cde^
Jimma wild	1.49 ± 0.55^p^	1.50 ± 0.19^tu^	28.53 ± 1.94^v^	2.83 ± 1.17^ab^
Jinka wild	4.04 ± 0.89^klm^	2.18 ± 0.25^q^	47.01 ± 2.95^p^	1.81 ± 0.41^def^
Malbec	7.83 ± 1.32^f^	4.30 ± 0.24^i^	83.57 ± 2.74^h^	1.33 ± 0.52^efghi^
Merlot	12.05 ± 1.68^c^	5.42 ± 0.19^d^	114.55 ± 4.26^d^	0.00 ± 0.00^l^
Perlette	3.84 ± 0.75^lm^	2.62 ± 0.17^o^	55.18 ± 2.96^n^	1.50 ± 0.57^efgh^
Pinot Noir	11.17 ± 1.47^cd^	5.13 ± 0.23^e^	114.30 ± 2.73^d^	0.52 ± 0.54^jkl^
Ruby seedless	5.81 ± 0.98^hij^	2.78 ± 0.24^n^	60.65 ± 3.96^l^	1.36 ± 1.21^efghi^
Sauvignon Blanc	10.29 ± 1.21^d^	4.83 ± 0.28^g^	100.42 ± 3.54^f^	0.83 ± 0.75^hijk^
Semillon	7.69 ± 1.03^f^	4.00 ± 0.25^j^	78.38 ± 4.97^i^	1.52 ± 0.52^efgh^
Sugraone	5.48 ± 0.84^ij^	3.40 ± 0.23^l^	74.30 ± 3.49^j^	0.18 ± 0.41^kl^
Syrah	16.32 ± 2.07^a^	6.23 ± 0.27^a^	134.73 ± 4.23^a^	0.00 ± 0.00^l^
Thompson seedless	7.18 ± 0.98^fg^	3.57 ± 0.19^k^	79.35 ± 3.69^i^	0.00 ± 0.00^l^

Mean	6.76	3.40	71.64	1.27
CV	14.03	3.17	2.57	25.77
LSD	1.08	0.12	2.11	0.81
*P* value	^*∗∗∗*^	^*∗∗∗*^	^*∗∗∗*^	^*∗∗∗*^

Means within a column followed by the same letter(s) are not significantly different at 5% LSD test (^*∗∗∗*^*P* < 0.001, ^*∗∗*^*P* < 0.01, ^*∗*^*P* < 0.05, and ns = *P* > 0.05).

**Table 3:** The main effect of research site locations on grapevine trunk diameter, root length, number of flower clusters per vine, and number of suckers per vine.

Location	Trunk diameter	Root length	Flower cluster	Suckers
Dilla	3.59 ± 1.44^a^	74.24 ± 31.81^a^	7.40 ± 4.57^a^	1.08 ± 0.93^b^
Yirgacheffe	3.20 ± 1.45^b^	69.05 ± 31.14^b^	6.11 ± 3.85^b^	1.45 ± 1.26^a^
Mean	3.40	71.64	6.76	1.27
CV	3.17	2.57	14.03	25.77
LSD	0.03	0.56	0.29	0.22
*P* value	^*∗∗∗*^	^*∗∗∗*^	^*∗∗∗*^	^*∗∗∗*^

Means within a column followed by the same letter(s) are not significantly different at 5% LSD test (^*∗∗∗*^*P* < 0.001, ^*∗∗*^*P* < 0.01, ^*∗∗∗*^*P* < 0.05, and ns = *P* > 0.05).

## Data Availability

The data that support the findings of this study are available upon request to the corresponding author.
